# Book Reviews

**Published:** 1897-11

**Authors:** 


### BOOK REVIEWS.

A Manual of Medical Jurisprudence. By Alfred S. Taylor, M.D., Lecturer on Medical Jurisprudence and Chemistry in Guy's Hospital, London. New American edition of 1897 from the twelfth English edition. Thoroughly revised by Clark Bell, Esq., of the New York Bar. In one octavo volume of 831 pages, with 54 engravings and 8 full-page plates. Cloth, $4.50; leather, $5.50. Lea Brothers & Co., Publishers, Philadelphia and New York, 1897.

Taylor's Medical Jurisprudence has long been a classic, and for a number of years it has been an important and valued work of reference in the libraries of many practitioners. The fact that successive editions are rapidly exhausted is sufficient evidence that popular estimation of the work continues. The present edition contains forty-five pages more than the preceding, and in the additional pages we note an interesting discussion of the medico-legal relations of the red-blood corpuscle, with several handsome plates showing the relative sizes of these corpuscles in different mammals. Under the head of Medical Secrets the well-known case of Kitson *vs.* Playfair is briefly presented, in which the editor expresses his sympathies with the plaintiff. This edition also contains two and a half new pages (by the editor, Dr. Bell) upon the "Judicial Evolution as to Criminal Responsibility of Inebriates," and the editor has added an entire chapter on "Medico-Legal Surgery," including Military Surgery, Naval Surgery and Railway Surgery. There are also other additions which together succeed in bringing this standard work strictly up to date. A good work on Medical Jurisprudence is indispensable to a physician. We heartily recommend this one.

---

The Diseases of Women. A Handbook for Students and Practitioners. By J. Bland Sutton, F.R.C.S., London, and Arthur E. Giles, M.D., B.Sc., London. 115 Illustrations. W. B. Saunders, 925 Wanut street, Philadelphia. $2.50.

Gynecology is a department of medicine which is exceedingly prolific of literature. New works upon this important specialty make their appearance every year, each possessing its own special merits and each adapted to the needs of a particular class of medical men. If there be any "long-felt want" in this branch of literature, it is the want of a book which shall meet the needs of advanced students and young practitioners. A book which will be comprehensive and yet concise, which shall emphasize diagnosis and give clear and explicit directions for treatment, which shall give prominence to gynecological therapeutics and minor gynecological surgery in preference to major operations, which shall be addressed to the student and practitioner rather than to the specialist—such a book on the diseases peculiar to women is sure to meet with general acceptance from the class of men for whom it is intended. The text-book of Sutton and Giles meets these requirements. It covers the subject in much the same manner that a judicious teacher would adopt. It lays a solid foundation for future study, is elementary in character, presupposes no knowledge of the subject on the part of the reader, and is yet sufficiently explicit to form a satisfactory guide to practice. Taken all in all, we consider it the best manual on this subject that has appeared in recent years.

V. O. H.

---

HYPNOTISM. Its Application to Practical Medicine. By Otto Wetterstrand, M.D., Member of the Society of Swedish Physicians at Stockholm; Corresponding Member of the Society for Psychical Research, London, etc. Authorized Translation by Henrik G. Petersen, M.D., Boston. Published by G. P. Putnam's Sons, 27 West 23d street, New York.

This volume is in two parts; Part I. being the translated work of Wetterstrand on Hypnotism; Part II. being a series of European letters by Dr. Petersen on Hypno-Suggestion.

There must be an intrinsic difference between the denizens of the old world and those of the new; for up to the present such wonderful clinical benefits have not been achieved in this country through the agency of Hypnotism as is so graphically portrayed and pointedly stated by the European writers.

With Mr. Herbert Spencer we are ready to acknowledge "A soul of truth in all things erroneous, and a soul of goodness in things evil"; but to this moment we have failed to see, and signally failed to achieve, through the agency of Suggestive Therapeutics, the results so common across the water.

Not for an instant doubting the truth of our foreign brothers, or robbing the subject of one iota's value, we cannot agree to the claims of suggestive therapeutics, as inculcated by Bernheim, Von Krafft-Ebing, Charcot, Liebeault, and others.

This volume, though not as extensive as some others, is not without interest. While falling short of the great scientific exposition of truth, as convincing as truth should be, further trials on our own part is invited, and maybe in the future the claims of the ardent exponents of the hypnotic art may see us equally enthusiastic.

H. H.

---

A PRACTICAL TREATISE ON SEXUAL DISORDERS OF THE MALE AND FEMALE. By Robert W. Taylor, M.D., Clinical Professor of Venereal Diseases in the College of Physicians and Surgeons, New York. In one handsome octavo volume of 448 pages, with 73 illustrations and 8 plates in color and monochrome. Cloth, $3.00 net. Lea Brothers & Co., New York and Philadelphia.

This work is very complete, and one of extreme value to the genito-urinary specialist. The general practitioner will find it a valuable addition to his library. The chapters on the anatomy and physiology of the sexual organs are more complete than is usually found in works of this kind. The different forms of impotency, causation and treatment, are clearly set forth, and show the careful study the author has given the subject. Gonorrhea, as a cause of many sexual disorders in the male, has been carefully studied and fully written upon. The pathology of the diseases of the prostate, the influence of prostatic disease upon sexual life, is a chapter full of interest, containing valuable information. The author attributes many sexual disorders to masturbation, probably more than can be justly laid at the door of this vile habit. The chapters on "disorders of the female sexual organs" are interesting, but not as full as others in the work. The illustrations are nicely gotten up, and the publishers deserve credit for the excellent typographical work. Taken altogether, it is one of the most complete treatises on the subject published. W. L. C.

---

LECTURES ON THE ACTION OF MEDICINES. By T. Lauder Brunton, M.D., LL.D., F.R.S., Fellow of the Royal College of Physicians; Physician and Lecturer on Pharmacology and Therapeutics to St. Bartholomew's Hospital. The MacMillan Company, 66 Fifth Ave., New York. [Price, $4.00.]

This is a course of Lectures on Pharmacy and Therapeutics delivered at St. Bartholomew's Hospital during the summer of 1896.

The study of Pharmacology and Therapeutics is, generally, a dry, heavy, monotonous task; and one in consequence not sufficiently mastered by the majority of medical men; at the same time occupying, from the utilitarian standpoint, a most important position.

The man who can, or rather has vested the subject with intense interest and made it a subject of pleasurable study is truly to be thanked. Every lecture in this volume is teeming with knowledge and vast instruction, presented most lucidly and simply.

It would be impossible to commend one portion rather than another. All chapters are gems. The English is good, and the clinical and didactic instructions exquisitely presented, and the whole book makes delightful reading.

Without hesitation we recommend this volume to the student, practitioner, and professor, feeling sure, that one and all will reap knowledge, personal courage, and confidence after its careful study.

The publishers have, as they always do, made a neat and attractive volume. H. H.

---

THE LIVER OF DYSPEPTICS. Particularly the Cirrhosis Produced by Auto-Intoxication of Gastro-Intestinal Origin. By Dr. Emile Boix, Paris. Authorized Translation by Paul R. Brown, M.D., U. S. Army. Published by G. P. Putnam's Sons, 27 West 23d St., New York.

The author of this book believes that cirrhosis of the liver has been too generally attributed to the abuse of alcoholic drinks, and he desires to call attention to the number of cases of hypertrophied and atrophied livers for which alcohol cannot be held responsible. He seeks to establish the fact that, in addition to alcohol, and entirely independent of it, there is another cause which may play an active part in the production of sclerosis of the liver—auto-intoxication of gastro-intestinal origin. The sclerosis thus resulting from auto-intoxication the author calls *dyspeptic cirrhosis*, as distinguished from true *alcoholic cirrhosis*.

The work is divided into three parts. In the first the author discusses auto-intoxication of gastro-intestinal origin, enumerates the products of fermentation capable of exercising a pathogenic action, and recites the conditions which favor abnormal fermentations and the production of these poisons. In the second part comes the history of the dyspeptic liver in its two forms, congestion and cirrhosis. Here also the author considers the anatomical form of dyspeptic cirrhosis and its place among the scleroses of the liver. The third part is devoted to the relation of the author's personal experiments with some of the agents *a priori* suspected as capable of causing gastro-intestinal fermentation and intoxication, etc.

The work is exceedingly instructive and of great scientific value.

---

A CLINICAL MANUAL. A Guide to the Practical Examination of the Excretions, Secretions and the Blood, for the use of Physicians and Students. By Andrew MacFarlane, A.B., M.D., Instructor in Neurology and Diseases of the Chest in the Albany Medical College, Physician to St. Peter's Hospital, Out Patient Department. Published by G. P. Putnam's Sons, 27 W. 23d St., New York.

This is a splendid manual upon the subject of which it treats. It contains 134 pages, with 32 illustrations. It is divided into six parts—the examination of the urine, stomach contents, feces, blood, pathological fluids and pathogenic micro-organisms. The importance of a thorough knowledge of the chemical and microscopical examination of the urine in diseased conditions is evident to every physician. The author gives the various methods of such examinations in a concise but thorough manner. The chapters on the examination of stomach contents, feces, blood and pathological fluids, are very interesting and full of valuable information. The book is neatly printed, well bound and makes an attractive little volume.

W. L. C.

---

PRACTICAL TREATISE ON MATERIA MEDICA AND THERAPEUTICS. By Roberts Bartholow, M.D., LL.D., Emeritus Professor of Materia Medica, Therapeutics and Hygiene in the Jefferson Medical College, Philadelphia, etc. Ninth edition. Revised and enlarged. D. Appleton & Co., 72 Fifth Avenue, New York. $5.00.

Perhaps no book is more familiar to physicians, or more deservedly popular, than this. In its several editions it has been before the profession for twenty years, and many classes of students have ground out their knowledge of materia medica from its pages. Successive editions have kept pace with the progress of therapeutics and pharmacology. For the ninth edition such additions and alterations have been made as were necessary to dispose of the new material which the rapid development of pharmacology has contributed to the science and art of therapeutics. This edition is larger than its predecessor by forty-five pages.

The book continues to be one of the best, for both student and practitioner. There is none better.

---

"COLLIER'S WEEKLY," OCTOBER 28TH.

"Collier's Weekly," for the week ending October 28th, contains, as usual, a wealth of literary comment and artistic *causerie*. An editorial on the late Chas. A. Dana is particularly noteworthy as the tribute of one of his best-known associates of the *Sun*.

"Our Note-Book" is iridescent as ever with Saltus's sparkling phrases, lighted, as always, with incident and epigram. Edgar Fawcett's London letter deals with the literary gossip of the "Capital of Letters," and Julian Hawthorne touches with trenchant pen upon many crying evils of the day.

The illustrations are executed for the most part on wood, and are of rare artistic excellence. "Lawrence Clavering," by A. E. W. Mason, enters its second installment with interest unabated.

Altogether, "Collier's Weekly" is a type of what a high-class illustrated weekly should be: elegant and elevating, a force that makes for culture and civilization. We commend it heartily to our readers.

